# Histomorphometric Quantitative Evaluation of Long-Term Risedronate Use in a Knee Osteoarthritis Rabbit Model

**DOI:** 10.3389/fvets.2021.669815

**Published:** 2021-04-22

**Authors:** Silvia Fernández-Martín, Antonio González-Cantalapiedra, María Permuy, Mario García-González, Mónica López-Peña, Fernando Muñoz

**Affiliations:** ^1^Anatomy, Animal Production and Veterinary Clinical Sciences Department, Veterinary Faculty, Universidade de Santiago de Compostela, Lugo, Spain; ^2^Ibonelab S.L., Laboratory of Biomaterials, Lugo, Spain

**Keywords:** cartilage, histomorphometry, long-term therapy, osteoarthritis, rabbit model, risedronate, subchondral bone

## Abstract

Osteoarthritis (OA) treatment is a major orthopedic challenge given that there is no ideal drug capable to reverse or stop the progression of the OA. In that regard, bisphosphonates have been proposed as potential disease-modifying drugs due to their possible chondroprotective effect related to obtaining a greater subchondral bone quality. However, their effectiveness in OA is still controversial and additionally, there is little evidence focused on their long-term effect in preclinical studies. The aim of this study was to evaluate the risedronate quantitative effect on articular and subchondral periarticular bone by histomorphometry, in an experimental rabbit model in an advanced stage of OA. Twenty-four adult New Zealand rabbits were included in the study. OA was surgically induced in one randomly chosen knee, using the contralateral as healthy control. Animals were divided into three groups (*n* = 8): placebo control group, sham surgery group and risedronate-treated group. After 24 weeks of treatment, cartilage and subchondral femorotibial pathology was evaluated by micro-computed tomography (micro-CT) and undecalcified histology. The research results demonstrated that the experimental animal model induced osteoarthritic changes in the operated joints, showing an increased cartilage thickness and fibrillation associated with underlying subchondral bone thinning and decreased trabecular bone quality. These changes were especially highlighted in the medial tibial compartments as a possible response to surgical instability. Regarding the trabecular analysis, significant correlations were found between 2D histomorphometry and 3D imaging micro-CT for the trabecular bone volume, trabecular separation, and the trabecular number. However, these associations were not strongly correlated, obtaining more precise measurements in the micro-CT analysis. Concerning the long-term risedronate treatment, it did not seem to have the capacity to reduce the osteoarthritic hypertrophic cartilage response and failed to diminish the superficial cartilage damage or prevent the trabecular bone loss. This study provides novel information about the quantitative effect of long-term risedronate use on synovial joint tissues.

## Introduction

Osteoarthritis (OA) is a multifactorial and progressive disease of the synovial joints associated with dysfunction and pain. Morphologically, it is characterized by gradual articular cartilage deterioration, subchondral bone sclerosis, synovial inflammation and osteophyte formation (1). Although OA involves all the synovial joint tissues, both the articular cartilage layer and the subchondral periarticular bone seem to show the most severe impact of the disease (2). However, the exact relationship between their roles in the onset of OA still remain controversial (3, 4). Nevertheless, recent research has shown that the cartilage degeneration was strongly related and occurred in parallel to subchondral bone changes (5).

It is well-known that during the OA process, the subchondral bone compartment undergoes structural changes including, among others, a thickened subchondral plate and an increased bone turnover, which could affect the overlying hyaline cartilage in response to altered biochemical properties (6). Bisphosphonates (BPs) have therefore been proposed as potential disease-modifying drugs due to their modulator function of inhibiting the bone-resorption activity (3, 7). Experimental studies with animal models have shown positive chondroprotective effects of various BPs associated with obtaining a greater subchondral bone quality. Specifically, beneficial effects on both cartilage and bone compartment have been demonstrated, among others, with alendronate (8–12), zoledronic acid (13, 14), pamidronate (15), and risedronate (11, 16, 17). By contrast, other preclinical studies have shown no disease-modifying effect, failing to prevent the cartilage erosion (18–21) and the osteophyte formation (19, 21–23).

Animal models have been widely used in OA research and have a critical role in studying the articular structural changes and evaluating the therapy efficacy of several drugs. However, one should take into account that OA is a complex and heterogeneous disease and consequently, no single animal model is capable to represent all aspects of the pathology process (7, 24). Thus, numerous surgical models have been outlined in different animal species, mainly focusing on the synovial joint of the knee (25, 26). They are based on a combination of joint instability, altered load bearing and inflammation to induce the osteoarthritic changes (7, 27). Small animal models are thoroughly used in OA research due to their easy handling, housing availability, and low costs (25). Specifically, rabbits are one of the most used OA models in preclinical studies. The induced methods described in these studies included the anterior cruciate ligament transection (ACLT) with or without total or partial meniscectomy and medial and collateral ligament section, among others (28, 29). The ACLT surgical instability technique in skeletally mature rabbits has been demonstrated as a reproducible and efficacious model and is capable to reproduce the cartilage, synovial and subchondral bone changes associated with OA, similarly to those observed in human disease (29). Although some anatomical similarities with the human knee have been described, there are notable differences in cartilage composition and joint mechanics when comparing rabbits and humans (30). Rabbit cartilage is ~10 times thinner than human cartilage and shows higher chondrocyte density (31). Additionally, one should know the load-bearing pattern of the animal model used, because there are remarkable differences depending on the species of the selected animal. Concerning rabbits, they appear to show a marked load-bearing in lateral compartments, unlike other species such as rodents, guinea pigs, or humans. It has therefore been observed that surgical medial meniscectomy in rabbits results in slower and less severe degenerative joint changes compared with lateral surgical procedures, which probably make the medial interventions more suitable for therapeutic evaluations (25).

Regarding the different methods of assessment used in OA preclinical research, histological techniques continue to be the “Gold Standards” in evaluating the disease progression and the severity of the articular cartilage damage. However, there is a great variety of microscopic scoring evaluations which make the comparisons between studies challenging (26). Additionally, these types of histologic evaluations may be partially influenced by assessor's subjectivity and they only provide qualitative and partially quantitative information (32, 33). Technological advances related to two-dimensional (2D) image analysis systems allow gaining significant objectivity, precision and reproducibility in quantifying main histological parameters reflecting the OA features at the synovial joint tissues, including the subchondral bone component. As for the latter, histomorphometry appears to remain the most suitable technique for this purpose as it can be conducted on undecalcified samples, which improves the knowledge on the bone compartment (34–39). Regarding the non-invasive three-dimensional (3D) imaging techniques, magnetic resonance imaging (MRI) and micro-computed tomography (micro-CT) have proven to be accurate and suitable quantitative tools in the study of the histological OA features as cartilage damage and calcified tissues change (33, 40–42).

Although BPs have been largely studied for the past two decades, the evidence of their efficacy continues to be deficient and important differences between preclinical and clinical findings are still present. Additionally, there is a significant lack of evidence on evaluating their effectiveness on the long term, and only a few preclinical studies have investigated their effects after 6 months of treatment (20, 43). This manuscript focused on the histological modifying-effects of risedronate on hyaline cartilage and subchondral bone, after 24 weeks of treatment, considering its possible positive impact by reducing the periarticular bone turnover (11, 16, 17, 23, 44). In this line of research, the long-term risedronate effect on the synovial structures has recently been evaluated by macroscopically and decalcified histology assessments as well as micro-CT general analysis (45). However, on this occasion, a thorough 2D undecalcified histomorphometric protocol was used for the study of the quantitative cartilage and bone changes. Besides, we assessed the 3D site-specific microarchitecture fluctuations of the trabecular bone, comparing them with the results observed in the 2D histomorphometry. To the best of the authors' knowledge, there are no studies that focused on the stereological characterization and quantitative evaluation of the risedronate use on cartilage, subchondral bone plate and trabecular bone in the rabbit ACLT model in an advanced stage of OA.

The study is aimed at quantitatively determine whether the antiresorptive therapies such as risedronate, may act as osteoarthritic disease-modifying drugs in the long term. The goal was to gain further insight into the microscopic degenerative changes in the properties of the different cartilage layers and subchondral bone, their precise anatomical location, as well as the bisphosphonate role in the osteoarthritis rabbit preclinical model.

## Materials and Methods

### Experimental Animal Model

Twenty-four healthy adult male New Zealand White rabbits (Granja San Bernardo, Navarra, Spain) with a mean body weight of 5 kg were used in this study upon approval of the protocol by the Ethical Committee of the University of Santiago de Compostela (Spain) (Reference number: 01/16/LU-002). The *in vivo* procedures were conducted according to the Spanish and European Union regulations about care and use of research animals and this paper was written following the Animals in Research Reporting *In Vivo* Experiments (ARRIVE) guidelines (46). The animal housing and experimental procedures were conducted in the Animal Experimentation Facility of the University of Santiago de Compostela (Lugo, Spain) in ventilated and enriched individual rabbit cages (R-suite, Tecniplast, Varese, Italy) in a humidity- and temperature-controlled room kept on a regular 12 h light/dark cycle. Food and tap water were provided *ad libitum*. All efforts were made to minimize animals' pain and distress as well as to reduce the number of animals used. Environmental enrichment included supply of previously autoclaved herb, fresh fruit, paper rolls and wood sticks. All animals were monitored daily by accredited veterinarians trained in laboratory animal science, who checked their health status, body weight and signs of pain and discomfort. The rabbits were acclimated for 3 weeks prior to being used in the study.

The anesthetic protocol and the postoperative treatment was thoroughly described in a recent publication of our research group (45). Osteoarthritis was surgically induced by ACLT and partial medial meniscectomy achieving the instability of the knee joint. Only one randomly selected knee per animal was operated, using the contralateral as healthy control (HT). Eight out of the 24 included rabbits belonged to the sham operated control group. For sham surgery, a similar joint exposure was made in one of the knees but in this case the anterior cruciate ligament and medial meniscus were left intact. Post-operatively, animals were allowed to perform free activity without joint immobilization.

Treatments began 3 weeks after the OA induction and were administered over a 24-week period by blinded personnel. Rabbits were divided into three groups of eight animals each, according to a computer-generated randomization list, as follows: the treated group (RIS) received a 2.5 mg once-weekly oral dose of risedronate, whereas the untreated control group (CONT) and the surgery control group (SHAM) received only saline as vehicle (NaCl 0.9%). The animals were euthanised at 27 weeks post-surgery by a sodium pentobarbital injection in the lateral auricular vein (100 mg/kg IV, Dolethal, Vétoquinol, Madrid, Spain), after sedation with ketamine (25 mg/kg IM, Imalgène 1000, Merial, Toulouse, France) and medetomidine (50 μg/kg IM, Domtor, Esteve, Barcelona, Spain). As mentioned in a previous publication (45), the risedronate dosage used in the study corresponds to the human clinical doses recalculated according to the mean weight of the animals.

**Figure 1:**
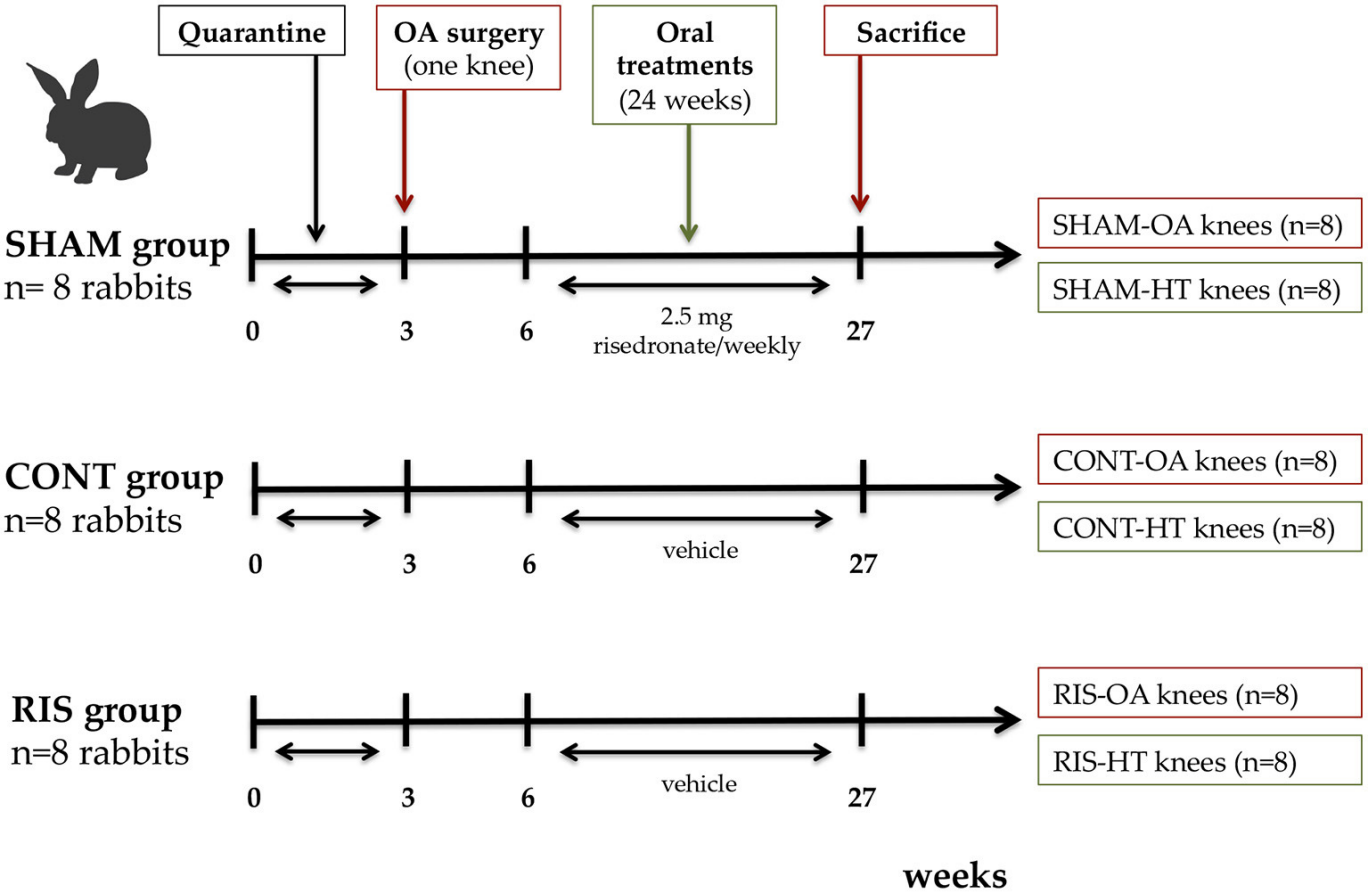
Diagram showing the schedule of the experimental design. SHAM group, surgery control group; CONT group, placebo treated group; RIS group, risedronate treated group; OA knee, operated osteoarthritic knee; HT knee, healthy contralateral knee.

Both knee joints were carefully dissected by sawing femora and tibias to characterize the progression of the osteoarthritic disease. The harvested knees were classified into the following six groups: (1) surgery control group-operated knees with placebo saline treatment (SHAM-OA, *n* = 8), (2) surgery control group-non-operated knees with placebo saline treatment (SHAM-HT, *n* = 8), (3) operated knees with placebo saline treatment (CONT-OA, *n* = 8), (4) non-operated knees with placebo saline treatment (CONT-HT, *n* = 8), (5) operated knees with risedronate treatment (RIS-OA, *n* = 8), and (6) non-operated knees with risedronate treatment (RIS-HT, *n* = 8) (Figure 1). Harvested specimens were preserved in 10% neutral buffered formalin and were destined for histomorphometry.

### Histology

#### Histological Preparation

Stifle joints were carefully dissected and standardized sections were obtained using a band saw from the middle region of each medial and lateral femoral condyle (MFC, LFC) and tibia plateau (MTP, LTP). Later, they were processed for undecalcified ground sections according to the method described by Donath (47). In summary, the fixed specimens were dehydrated in ascending grades of ethanol, and then infiltrated and embedded with a light curing methylmethacrylate resin (Technovit 7200-VLC, Heraus Kulzer GmbH, Werheim, Germany). Sections were cut and polished using a grinding machine (EXAKT Apparatebau, Norderstedt, Germany) up to ~40 μm in thickness and stained with Lévai-Laczkó.

#### Histomorphometric Analysis

Undecalcified histological samples were converted into digital format using a motorized light microscope containing a digital camera connected to a PC-based image capture system (BX51, DP71, Olympus Corporation, Japan). All the sections were observed and captured at magnifications up to x4. The microscopic histomorphometric evaluation was performed according to a previous publication of our research group (16). For that purpose, we used the following PC-based image analysis programs: Cell-sens 1.5 (Olympus Corporation, Japan) and Image-Pro Premier 9.0.4 (Media Cybernetics, Bethesda, MD, USA). All samples were presented in random order and were processed and evaluated by two experienced observers, blinded to the treatment received and the rabbit group. The cartilage and subchondral bone thickness, the fibrillation index and the trabecular subchondral bone area analysis were evaluated as follows.

**Figure 2:**
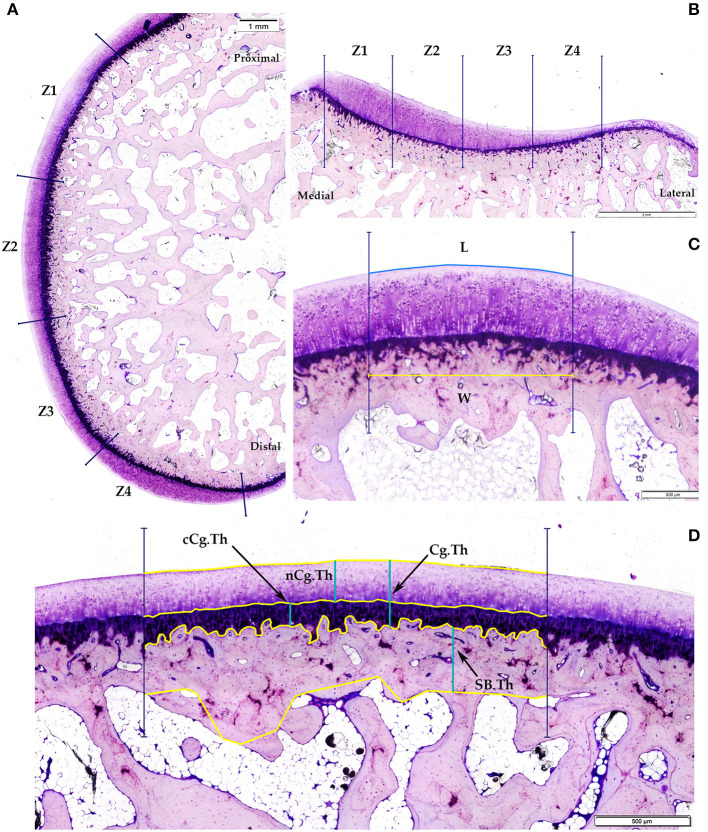
Representative images of histomorphometric analysis. **(A)** Femoral condyle ROI; **(B)** Tibial plateau ROI; **(C)** Fibrillation index (FI) calculated as the difference between the length of the cartilage surface (L) and a straight parallel with the same width line (W); **(D)** Cartilage and subchondral bone thickness: cCg.Th, non-calcified cartilage thicknes; cCg.Th, calcified-cartilage thickness; Cg.Th, total cartilage thickness; SB.Th, subchondral bone cortical thickness.

Firstly, a standardized region of interest (ROI) was defined at the margins of cartilage, located superiorly and inferiorly in the femur condyles, as well as laterally and medially in the tibial plateaus. The established ROI was divided into four equal distance zones (Z1 to Z4) with the purpose of reliably represent the total extent of the lesions on these surfaces. At each area, the cartilage surface and the junction between the uncalcified cartilage and calcified cartilage, corresponding to the tidemark line, were drawn. Furthermore, the junction points between the calcified cartilage and the subchondral bone plate and between the latter and the epiphyseal trabeculae bone initiation were identified and traced (Figure 2).

The following measurements of cartilage and subchondral bone thickness were calculated separately from the four histological zones of the ROI from each lateral and medial femorotibial joint compartments and expressed as mean distances:

(A) Non-calcified cartilage thickness (nCg.Th, μm): as the mean distance between the articular cartilage surface and the tidemark.
(B) Calcified cartilage thickness (cCg.Th, μm): as the mean distance from the tidemark to the subchondral bone plate.
(C) Total cartilage thickness (CgTh, μm): as the sum of the thickness of uncalcified and calcified layer.
(D) Subchondral bone cortical thickness (SB.Th, μm): as the mean distance between the subchondral bone plate and the trabecular bone initiation.

Additionally, the degree of the superficial fissures and surface undulations of the upper cartilage was quantified in all four zones of the femur and tibial compartments. This fibrillation index (FI) was assessed and calculated as the difference between the length of the cartilage surface (L) and a straight parallel with the same width line (W) (Figure 2).

**Figure 3:**
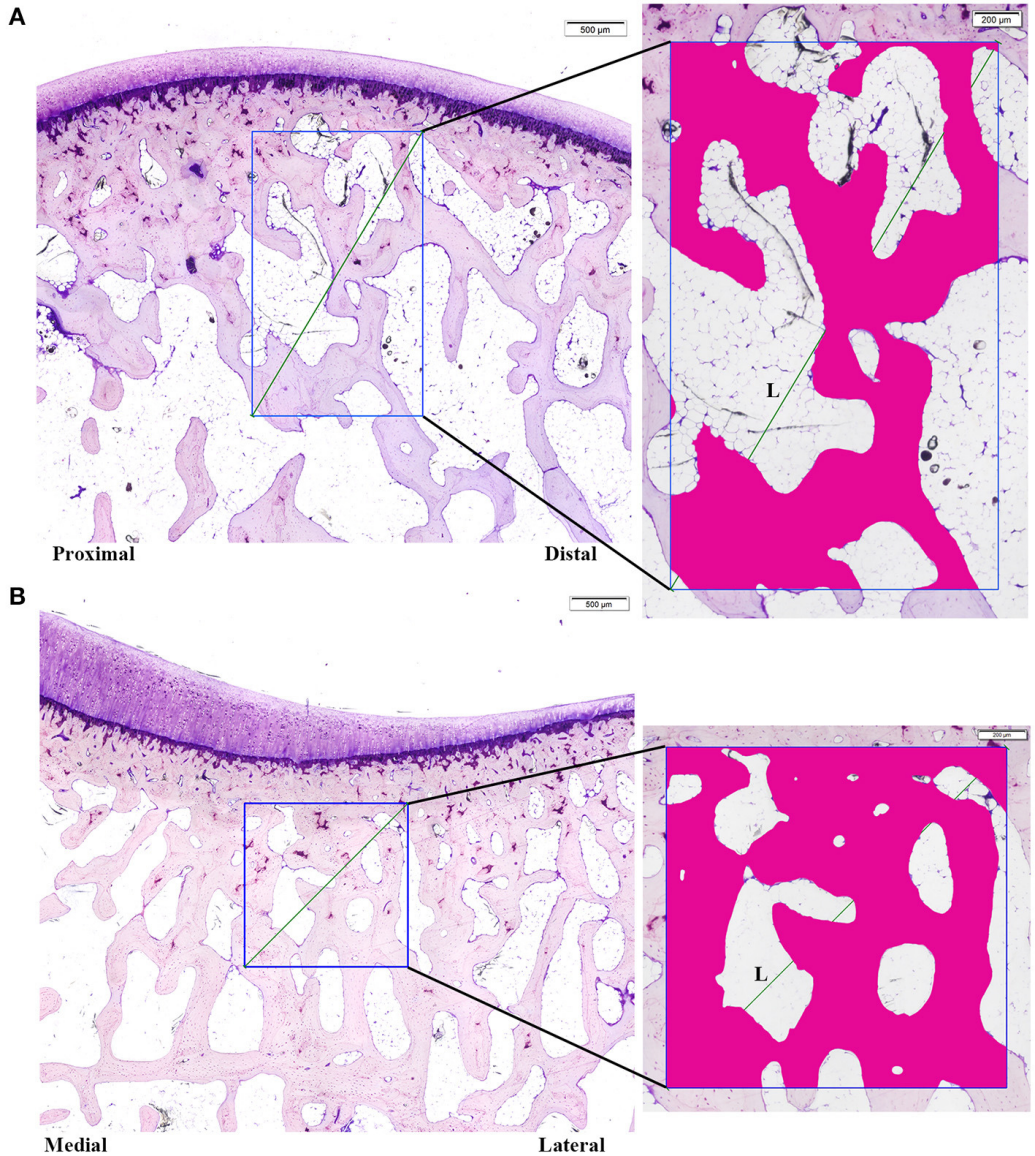
Trabecular subchondral bone histology measurements. **(A)** Femoral ROI (1.5 × 2.5 mm) **(B)** Tibial ROI (1.5 × 1.5 mm). Tb.A: % of trabecular bone in the ROI; Tb.Sp measured on the diagonal of the ROI [Tb.Sp = (L/Tb.N)-Tb.Th].

Concerning the trabecular analysis, the ROIs were defined on the central midpoint of the femur and tibia digital images including the subchondral trabecular bone underlying the articular cartilage with a size of 2.5 × 1.5 mm for the femur samples and 1.5 × 1.5 mm for the tibias (Figure 3). The trabecular subchondral bone parameters evaluated were: Trabecular area (Tb.A, %), trabecular thickness (Tb.Th, mm), trabecular separation (Tb.S, mm), and trabecular number (Tb.N, 1/mm).

### Micro-Computed Tomography Evaluation

**Figure 4:**
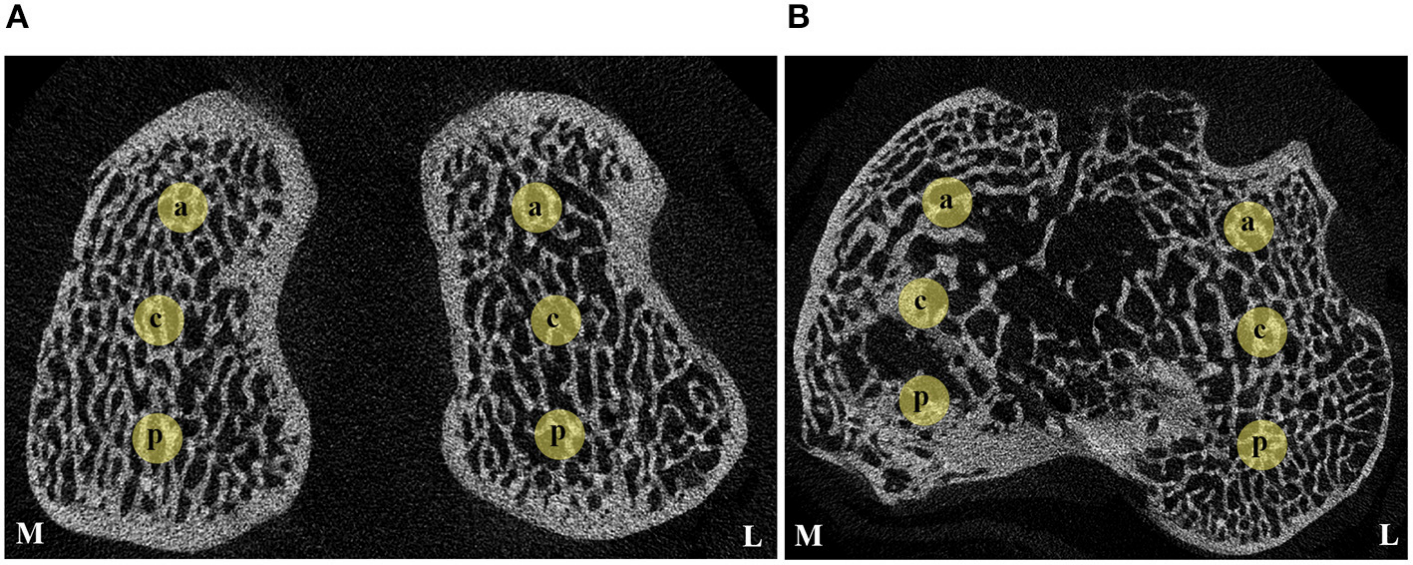
Transverse micro-CT slices indicating the anatomical VOIs of cancellous bone evaluated. **(A)** Distal femur; **(B)** Proximal tibia. M, medial; L, lateral; a, anterior; c, central; p, posterior.

In order to measure the 3D structural parameters of the trabecular bone, the distal part of the femur and proximal part of the tibia were scanned with a high-resolution micro-CT (Skyscan 1172, Bruker microCT NV, Kontich, Belgium) using parameters according to our previous study (45). In order to evaluate the specific regional changes and based on the method described by Batiste et al. (40), the knee was examined at the lateral and medial sides of femoral condyles and tibial plateaus and three cylindrical volumes of interest (VOIs), of ×2.5 mm of diameter and 2.5 mm depth for femoral compartments and 2.5 × 1.5 mm for tibial compartments, were placed in separate anatomical regions: anterior, central and posterior (Figure 4). Additionally, the central VOIs of both femoral and tibial samples were compared and correlated with the previous 2D histologic findings of the trabecular bone. The following indices of cancellous bone microarchitecture were evaluated: bone volume fraction (BV/TV, %), trabecular thickness (Tb.Th, mm), trabecular separation (Tb.S, mm), and trabecular number (Tb.N, 1/mm).

### Statistical Analysis

Data were expressed as means ± standard deviations (SDs). All analyses were performed using SigmaPlot 12.5 software (Systat Software inc., Chicago, IL, USA). The normality of the data was assessed using the Shapiro-Wilk test and Levene's test was employed to assess the equality of variances of normal variables. The differences between the two groups were tested for significance by Student's *t*-test or by two-tailed Mann-Whitney *U*-test when data did not follow a normal distribution. For statistical analysis among the three different groups, means were compared using a one-way ANOVA and by applying a *post-hoc* Holm-Sidak test. For non-normal variables, the statistical comparison was performed using the Kruskal-Wallis *H*-test and the *post-hoc* analysis using Dunn's test. Pearson's correlation analyses were performed between the undecalcified histology trabecular parameters and the micro-CT measurements in the entire sample. *P* < 0.05 was considered statistically significant.

## Results

### Animals

During the experimental procedures no changes in weight or general condition were observed. All surgeries were performed without complications. The animals successfully tolerated the treatments and no adverse reactions to risedronate or vehicle were noted. Only one rabbit belonging to the SHAM group died suddenly due to an unknown reasons.

### Histomorphometric Results

A total of 184 histological blocks were obtained. Out of these, five were excluded from the histomorphometric analysis due to excessive slicing artifact or incorrect anatomical section, whereas 179 of them were finally included for assessment. Results can be summarized in three points.

#### Cartilage and Subchondral Bone Cortical Thickness

Histomorphometric analysis data of the cartilage and subchondral bone thickness for undecalcified femoral condyles and tibial plateaus are shown in Tables 1, 2 respectively.

**Table 1:** Stereologic histomorphometric analysis of femoral condyles.

			**nCg.Th (μm)**		**cCg.Th (μm)**		**Cg.Th (μm)**		**SB.Th (μm)**	
		**Zones**	**LFC**	**MFC**	**LFC**	**MFC**	**LFC**	**MFC**	**LFC**	**MFC**
Osteoarthritis joints (OA)	SHAM	1	308.90 ± 94.56	330.86 ± 98.76	155.99 ± 64.04	165.52 ± 41.11	462.96 ± 116.13	495.34 ± 125.70	327.34 ± 117.29	257.53 ± 84.60
		2	324.88 ± 74.29	365.82 ±80.27	153.15 ± 28.41	193.63 ± 47.70	474.91 ± 90.49	557.12 ± 91.03	400.54 ± 125.70	370.94 ± 86.77
		3	329.37 ± 94.66	415.71 ± 99.98	120.18 ± 22.60	137.74 ± 28.03	446.10 ± 103.23	506.05 ± 132.71	464.52 ± 182.24	390.99 ± 124.91
		4	318.09 ± 99.83	406.77 ± 103.82	105.39 ± 27.51	124.60 ± 33.33	423.14 ± 106.76	530.56 ± 105.02	254.97 ± 82.21	343.54 ± 84.17
	CONT	1	**519.96** **±** **206.74**^**a**^	481.48 ± 126.99	156.13 ± 57.80	178.30 ± 55.83	679.75 ± 240.83	658.65 ± 161.95	216.44 ± 92.95	247.97 ± 80.33
		2	500.51 ± 221.56	453.46 ± 128.61	182.99 ± 69.47	172.87 ± 65.37	685.02 ± 278.19	620.49 ± 185.88	331.67 ± 167.10	269.48 ± 98.21
		3	483.63 ± 119.97	421.07 ± 164.20	159.25 ± 73.16	114.58 ± 36.88	638.70 ± 179.83	530.08 ± 174.27	280.06 ± 154.66	317.07 ± 98.58
		4	**576.74** **±** **147.42**^**a**^	**390.96** **±** **176.58**^**+**^	139.42 ± 39.04	**94.20** **±** **24.14**^**+**^	**714.92** **±** **153.58**^**a**^	**480.58** **±** **191.40**^**+**^	169.17 ± 99.66	262.29 ± 138.16
	RIS	1	**555.21** **±** **87.53**^**a**^	508.75 ± 203.74	150.57 ± 49.70	185.78 ± 60.75	695.67 ± 115.65	686.94 ± 250.60	267.65 ± 77.55	312.61 ± 125.55
		2	477.58 ±155.29	501.66 ± 184.39	183.91 ± 58.57	223.89 ± 69.80	655.04 ± 206.34	722.64 ± 167.32	332.99 ± 104.37	256.35 ± 96.83
		3	442.43 ±180.42	**637.37** **±** **189.86**^**a**^^**,**^ ^**b**^	143.44 ± 81.24	169.28 ± 61.28	581.91 ± 253.02	**801.84** **±** **224.31^a^^,^^b^**	320.39 ± 92.21	**241.98** **±** **79.14**^**a**^
		4	**503.73** **±** **81.70**^**a**^	555.99 ± 104.28	151.74 ± 60.20	135.82 ± 46.21	**647.00** **±** **123.13**^**a**^	**684.50** **±** **119.92**^**a**^	223.49 ± 52.11	306.57 ± 94.30
Healthy joints (HT)	SHAM	1	**187.96** **±** 51.80^*^	**345.11** **±** **92.90**^**+**^	93.50 ± 33.56	**157.47** **±** **47.32**^**+**^	**279.78** **±** 81.84^*^	**499.57** **±** **107.12**^**+**^	357.18 ± 50.95	270.02 ± 120.21
		2	298.93 ± 42.93	375.193 ± 101.50	139.71 ± 35.01	179.88 ± 38.21	436.22 ± 60.13	551.63 ± 132.76	412.94 ± 88.09	414.38 ± 182.44
		3	275.33 ± 67.19	**415.01** **±59.70**^**+**^	114.84 ± 20.94	136.18 ± 27.35	385.43 ± 77.95	**547.17** **±** **83.06**^**+**^	381.37 ± 131.04	398.48 ± 99.51
		4	273.85 ± 104.86	**395.309** **±** **94.28**^**+**^	91.46 ± 13.61	**110.76** **±** **14.67**^**+**^	363.94 ± 107.23	**502.66** **±** **99.82**^**+**^	289.10 ± 130.79	410.66 ± 110.49
	CONT	1	**270.71** **±** 112.06^*^	352.038 ± 142.82	127.27 ± 31.66	136.82 ± 44.53	**392.74** **±** 118.67^*^	**486.68** **±** 157.12^*^	293.45 ± 70.42	261.75 ± 121.34
		2	337.71 ± 105.37	**307.72** ±105.11^*^	154.01 ± 51.17	148.29 ± 33.92	488.72 ± 151.53	473.86 ± 101.51	315.12 ± 69.24	307.66 ± 114.08
		3	**311.21** **±** 81.48^*^	389.43 ±71.77	119.68 ± 35.47	107.27 ± 18.16	**427.00** **±** 115.35^*^	491.58 ± 72.94	377.62 ± 118.35	353.68 ± 94.66
		4	**301.60** **±** 119.09^*^	378.59 ± 76.42	103.76 ± 20.98	98.21 ± 21.52	**383.28** **±** 136.76^*^	474.60 ± 101.13	**330.52** **±** 124.97^*^	363.32 ± 124.07
	RIS	1	**267.70** **±** 95.34^*^	**365.59** **±** **29.61**^**+**^	124.91 ± 34.24	144.53 ± 17.25	**387.02** **±** 118.90^*^	505.33 ± 24.50	346.58 ± 88.60	283.84 ± 92.94
		2	**326.144** **±** 105.42^*^	373.23 ± 42.66	139.70 ± 37.32	167.99 ± 24.86	460.85 ± 137.65	**536.94** **±** 65.82^*^	329.63 ± 150.10	**378.58** **±** 99.57^*^
		3	318.97 ± 125.91	**369.63** **±** 56.93^*^	117.78 ± 37.60	120.38 ±17.54	431.95 ± 160.30	**483.11** **±** 59.60^*^	367.75 ± 84.70	**395.36** **±** 80.62^*^
		4	**299.68** **±** 119.45^*^	**400.02** **±** 89.15^*^	114.78 ± 25.32	85.28 **±7.08**^a^^,^^+^,^*^	**411.29** **±** 141.35^*^	**478.52** **±** 91.36^*^	291.06 ± 80.93	374.99 ± 145.58

Hismorphometric results: LFC, lateral femoral condyle; MFC, medial femoral condyle, nCg.Th, non-calcified cartilage thickness; cCg.Th, calcified cartilage thickness; Cg.Th, total cartilage thickness; SB.Th, subchondral bone thickness. Values represent the mean and SD. Statistical significant differences are marked in “bold text.” p < 0.05:

a *vs. SHAM*,

b *vs. CONT*,

+ *vs. lateral compartment*,

* *vs. OA joint*.

**Table 2:** Stereologic histomorphometric analysis of tibial plateaus.

			**nCg.Th (μm)**		**cCg.Th (μm)**		**Cg.Th (μm)**		**SB.Th (μm)**	
		**Zones**	**LTP**	**MTP**	**LTP**	**MTP**	**LTP**	**MTP**	**LTP**	**MTP**
Osteoarthritis joints (OA)	SHAM	1	477.85 ± 87.59	651.52 ± 239.86	132.71 ± 27.66	**91.97** **±** **24.13**^**+**^	610.60 ± 82.71	739.17 ± 236.18	427.174 ± 88.81	450.75 ± 71.12
		2	516.90 ± 88.22	816.82 ± 284.96	89.06 ± 20.43	**71.08** **±** **7.07**^**+**^	600.96 ± 75.35	878.18 ± 275.86	458.98 ± 54.92	529.07 ± 130.33
		3	273.57 ± 42.13	**658.05** **±** **203.22**^**+**^	79.90 ± 10.86	72.52 ± 19.39	350.39 ± 43.55	731.08 ± 196.87	317.31 ± 107.64	**438.12** **±** **65.29**^**+**^
		4	154.02 ± 19.43	**370.04** **±** **129.92**^**+**^	80.32 ± 20.40	100.53 ± 27.12	232.41 ± 32.61	**470.20** **±** **139.73**^**+**^	173.11 ± 65.72	**347.09** **±** **74.64**^**+**^
	CONT	1	593.95 ± 310.96	782.83 ± 279.39	137.28 ± 60.04	**82.45** **±** **29.86**^**+**^	735.34 ± 329.55	866.93 ± 253.20	**302.43** **±** **104.14**^**a**^	**489.89** **±** **109.80**^**+**^
		2	656.44 ± 186.21	**974.09** **±** **97.23**^**+**^	120.49 ± 49.42	**57.11** **±** **10.70**^**+**^	772.27 ± 173.82	**1024.82** **±** **97.94**^**+**^	**289.07** **±** **116.07**^**a**^	368.61 ± 65.58
		3	**529.06** **±** **152.64**^**a**^	**740.43** **±** **188.85**^**+**^	133.90 ± 85.47	**61.51** **±** **21.26**^**+**^	**668.31** **±** **97.68**^**a**^	796.80 ± 209.01	235.30 ± 62.88	**321.82** **±** **72.56^a^^,^^+^**
		4	**362.98** **±** **22.87**^**a**^	553.92 ±125.38	90.03 ± 34.99	107.45 ± 35.75	450.37 ± 246.42	650.23 ± 131.00	166.96 ± 102.31	**393.51** **±** **93.84**^**+**^
	RIS	1	585.26 ± 268.53	680.26 ± 210.67	131.93 ± 29.77	**125.34** **±** **64.07**^**b**^	710.52 ± 285.12	831.21 ± 202.79	384.59 ± 73.25	440.46 ± 167.95
		2	567.66 ± 245.37	804.24 ± 284.64	101.72 ± 42.97	**91.78** **±** **33.42**^**b**^	659.43 ± 244.29	888.42 ± 272.78	373.51 ± 84.08	413.30 ± 141.63
		3	419.49 ± 142.92	**653.24** **±** **224.08**^**+**^	108.92 ± 40.63	**95.67** **±** **39.28**^**b**^	**522.68** **±** **132.44^a^^,^^b^**	742.22 ± 194.12	258.47 ± 84.88	354.66 ± 133.07
		4	**276.14** **±** **146.47**^**a**^	**503.90** **±** **178.04**^**+**^	80.51 ± 24.53	**141.45** **±** **65.23**^**+**^	349.39 ± 163.45	**638.52** **±** **160.86**^**+**^	220.39 ± 90.87	**461.08** **±** **257.66**^**+**^
Healthy joints (HT)	SHAM	1	421.83 ± 95.72	**718.42** **±** **165.38**^**+**^	133.76 ± 32.92	**87.53** **±** **14.26**^**+**^	553.27 ± 88.79	**808.22** **±** **166.26**^**+**^	491.91 ± 294.44	499.98 ± 54.85
		2	436.50 ±128.13	**1034.55** **±** **113.86**^**+**^	93.74 ± 24.70	69.89 ± 13.39	525.42 ± 130.22	**1104.39** **±** **112.50**^**+**^	475.91 ± 186.53	494.55 ± 53.90
		3	238.12 ± 76.55	**682.82** **±** **54.87**^**+**^	76.53 ± 23.41	63.23 ± 19.50	308.54 ± 89.78	737.32 ± 50.49	337.79 ± 118.75	**409.40** **±** **15.31**^**+**^
		4	145.04 ± 20.02	**425.05** **±** **67.06**^**+**^	67.49 ± 10.36	**84.55** **±** **16.23**^**+**^	208.44 ± 29.61	**507.48** **±** **59.71**^**+**^	192.37 ± 65.41	**394.38** **±** **81.84**^**+**^
	CONT	1	419.67 ± 151.53	**767.16** **±** **107.28**^**+**^	127.93 ± 31.99	**71.07** **±** **9.27**^**+**^	545.38 ± 136.55	**840.47** **±** **96.31**^**+**^	392.47 ± 63.95	421.64 ± 87.43
		2	**440.49** **±** 182.26^*^	845.86 **±108.91**^+^,^*^	94.43 ± 19.75	**66.39** **±** **23.98**^**+**^	**527.80** **±** 167.95^*^	904.53 **±103.87**^+^,^*^	334.67 ± 65.4	395.35 ± 39.55
		3	**249.20** **±** 138.75^*^	**594.94** **±** **107.93**^**+**^	79.75 ± 10.10	80.84 ± 31.63	**322.68** **±** 135.03^*^	672.21 ± 104.05	267.06 ± 27.61	360.44 ± 107.74
		4	**141.05** **±** 44.82^*^	366.88 **±84.21**^+^,^*^	83.28 ±15.92	106.79 ± 39.36	**223.34** **±** 41.38^*^	472.43 **±65.06**^+^,^*^	180.04 ± 44.90	**326.89** **±** **80.28**^**+**^
	RIS	1	533.57 ± 242.19	688.92 ± 220.71	113.86 ± 31.79	**95.24** **±** 36.13^*^	649.99 ± 236.22	765.12 ± 199.43	496.55 ± 160.81	551.99 ± 180.55
		2	456.03 ± 171.77	**975.63** **±** **220.71**^**+**^	89.28 ± 15.88	99.35 ± 48.59	537.55 ± 172.79	**1075.03** **±** **364.61**^**+**^	466.38 ± 120.40	**545.6** **±** **112.96**^**b**^
		3	**263.61** **±** 109.89^*^	**700.06** **±** **255.60**^**+**^	**75.67** **±** 11.57^*^	78.20 ± 37.76	**334.56** **±** 116.09^*^	773.25 ± 236.94	437.28 **±112.07**^b^,^*^	**464.89** **±** 69.86^*^
		4	**137.53** **±** 44.01^*^	**406.59** **±** **150.94**^**+**^	81.32 ± 20.11	92.25 ± 46.79	**216.84** **±** 58.59^*^	501.60 **±147.15**^+^,^*^	**259.82** **±** **40.98^a^^,^^b^**	**362.35** **±** **91.63**^**+**^

Hismorphometric results: LTP, lateral tibial plateau; MTP, medial tibial plateau, nCg.Th, non-calcified cartilage thickness; cCg.Th, calcified cartilage thickness; Cg.Th, total cartilage thickness; SB.Th, subchondral bone thickness. Values represent the mean and SD. Statistical significant differences are marked in “bold text.” p < 0.05:

a *vs. SHAM*,

b *vs. CONT*,

+ *vs. lateral compartment*,

* *vs. OA joint*.

Regarding the cartilage parameters analyzed in the femoral and tibial compartments, we observed a significant increase of the Cg.Th in the injured knees corresponding to CONT-OA and RIS-OA animals vs. their contralateral healthy joint. Furthermore, a markedly increased Cg.Th was observed at the medial tibial sides in both injured and not injured knees. Regarding the cartilage areas, the highest values were shown in Z1 and Z2 in all the examined tibias.

The separate analysis of the layers of cartilage of femoral condyles and tibial plateaus, revealed that the uncalcified layer showed significantly higher thickness values in the osteoarthritic joints compared to the healthy ones, in agreement with what was observed in the Cg.Th analysis. Once again, MTP samples and tibial Z1 and Z2 revealed the higher thickness measurements. With regard to the calcified level, small differences in the statistical analysis were observed between groups. However, RIS-OA and CONT-OA appeared to show a little tendency toward thickening of the calcified cartilage, with the exception of the observed in the CONT-OA group of the medial tibial compartment, where a marked reduction in cCg.Th was outlined.

In summary, osteoarthritic joints showed the highest thickness values, as well as MTP in all the evaluated knees. Additionally, risedronate treatment did not have the capacity to prevent the cartilage thickening and no significant differences were found when comparing with the untreated-control group.

As for the SB.Th, in the analysis of the femoral condyles, no significant differences were found between risedronate-treated animals and untreated-control groups (SHAM and CONT) and neither between the lateral and medial condyles. However, we noticed that the osteoarthritic joints (CONT-OA and RIS-OA) showed the lowest values of all the groups and these differences were significant in some of the areas analyzed when comparing with HT joints. With respect to tibial plateaus, the main statistical difference was found between the lateral and medial compartment, showing significant higher thickness values at the MTP in both OA and HT joints. Regarding the separate analysis of healthy groups, risedronate administration showed a tendency to increase the SB.Th values compared to untreated rabbits (CONT-HT and SHAM-HT). As observed in terms of cartilage thickness, Z1 and Z2 generally revealed the highest thickness measurements in all the tibial analyzed groups.

### Fibrillation Index

The results of the FI evaluation are summarized in Table 3. Regarding the degree of superficial fissures and surface undulations of articular cartilage, CONT-OA and RIS-OA animals showed the highest values in all the compartments and all the evaluated zones. It is therefore important to note that risedronate therapy did not seem to improve the cartilage pathological changes and no significant differences were found when comparing placebo control and risedronate-treated OA groups. As far as the location is concerned, medial compartments seemed to show more surface fibrillation than lateral compartments. However, in relation to the results when analyzing the four included successive zones, the values were too variable to draw any conclusions. Regarding HT joints, we noticed that the values were slightly higher in the medial compartments compared to the lateral ones, especially in the medial tibial side. Additionally, although we found no significant differences between SHAM-HT and SHAM-OA groups, a slight increase in the FI scores was observed in the osteoarthritic sham animals in comparison to the healthy ones.

**Table 3:** Fibrillation index in the four evaluated zones (Z1 to Z4).

		**Osteoarthritis joints (OA)**				**Healthy joints (HT)**			
		**Z1**	**Z2**	**Z3**	**Z4**	**Z1**	**Z2**	**Z3**	**Z4**
SHAM	LFC	1.040 ± 0.018	1.030 ± 0.024	1.047 ± 0.053	1.086 ± 0.055	**1.020** **±** 0.010^*^	1.010 ± 0.010	1.030 ± 0.010	1.051 ± 0.024
	MFC	1.052 ± 0.028	1.024 ± 0.031	1.104 ± 0.102	1.110 ± 0.063	**1.043** **±** **0.031**^**+**^	**1.034** **±** **0.025**^**+**^	**1.069** **±** **0.037**^**+**^	1.081 ± 0.041
	LTP	1.045 ± 0.093	1.011 ± 0.019	1.011 ± 0.007	1.021 ± 0.041	1.011 ± 0.015	1.010 ± 0.016	1.010 ± 0.009	1.040 ± 0.051
	MTP	1.068 ± 0.064	**1.194** **±** **0.233**^**+**^	1.086 ± 0.112	1.066 ± 0.070	**1.084** **±** **0.054**^**+**^	**1.100** **±** **0.040**^**+**^	1.094 ± 0.045	1.090 ± 0.060
CONT	LFC	1.040 ± 0.019	1.042 ± 0.054	1.059 ± 0.080	1.080 ± 0.033	1.032 ± 0.027	1.019 ± 0.012	1.031 ± 0.020	**1.044** **±** 0.024^*^
	MFC	**1.088** **±** **0.050**^**+**^	**1.153** **±** **0.185^a^^,^^+^**	1.150 ± 0.170	**1.141** **±** **0.051**^**+**^	**1.033** **±** 0.025^*^	**1.015** **±** 0.011^*^	**1.030** **±** 0.015^*^	**1.044** **±** 0.008^*^
	LTP	1.111 ± 0.095	1.152 ± 0.241	1.073 ± 0.095	1.092 ± 0.094	1.025 ± 0.039	1.020 ± 0.019	1.024 ± 0.021	**1.006** **±** 0.006^*^
	MTP	1.179 ± 0.147	**1.313** **±** **0.251**^**+**^	1.234 ± 0.114	**1.262** **±** **0.146**^**a**^	**1.088** **±** **0.050**^**+**^	**1.105** **±** **0.064**^**+**^	**1.070** **±** 0.074^*^	**1.067** ±0.048^*^
RIS	LFC	1.201 ± 0.170	1.133 ± 0.105	1.158 ± 0.120	1.126 ± 0.045	**1.030** **±** 0.022^*^	1.038 ± 0.036	**1.025** **±** 0.009^*^	**1.058** **±** 0.040^*^
	MFC	1.170 ± 0.179	**1.156** **±** **0.077**^**a**^	1.193 ± 0.176	1.195 ± 0.136	1.016 **±0.012**^a^,^*^	**1.029** **±** 0.031^*^	**1.073** **±** **0.062**^**+**^	**1.075** **±** 0.048^*^
	LTP	1.147 ± 0.166	1.094 ± 0.149	1.083 ± 0.067	1.088 ± 0.141	**1.023** **±** 0.040^*^	**1.013** **±** 0.025^*^	**1.026** **±** 0.033^*^	**1.010** **±** 0.022^*^
	MTP	1.136 ± 0.157	1.292 ± 0.252	1.241 ± 0.278	1.156 ± 0.099	1.066 ± 0.066	**1.095** **±** **0.074**^**+**^	1.085 ± 0.048	**1.033** **±** 0.043^*^

LFC, lateral femoral condyle; MFC, medial femoral condyle; LTP, lateral tibial plateau; MTP, medial tibial plateau. Values represent the mean and SD. Statistical sifnificant differences are marked in “bold text.” p < 0.05:

a *vs. SHAM*,

+ *vs. lateral compartment*,

* *vs. OA joint*.

### Trabecular Subchondral Bone Analysis

Regarding the trabecular parameters evaluated by 2D histomorphometry (Table 4), there were no statistical significant differences in any of the parameters measured in the femoral compartments. Concerning tibial samples, the only significant differences were found in the Tb.A when comparing CONT-OA and CONT-HT as well as, CONT-OA and SHAM-OA, showing lower values in the injured animals. Additionally, we noticed that lateral tibial compartment exhibited the highest values in all HT and OA joints. No significant differences were found in the evaluation of the Tb.Th and Tb.Sp among groups. However, the LTP showed less trabecular separation than that observed in the other synovial compartments. Tb.N measurements were also not statistically significant between treated and untreated-groups, but significant decreased values were found when comparing the medial side against the lateral side and also, when comparing CONT-OA with CONT-HT groups at the MTP.

**Table 4:** Histomorphometrical trabecular analysis of healthy and OA joints.

		**Osteoarthritis joints (OA)**			**Healthy joints (HT)**		
		**SHAM**	**CONT**	**RIS**	**SHAM**	**CONT**	**RIS**
Tb.A (%)	Lateral femur	42.57 ± 5.15	49.57 ± 11.26	41.82 ± 8.46	46.76 ± 6.90	45.62 ± 5.77	47.54 ± 5.84
	Medial femur	45.32 ± 3.77	44.58 ± 6.23	46.11 ± 11.04	50.95 ± 9.79	50.20 ± 9.18	50.74 ± 3.84
	Lateral tibia	62.05 ± 3.70	**48.99** **±** **6.19**^**a**^	54.74 ± 8.96	62.69 ± 5.36	**61.62** **±** 2.45^*^	58.24 ± 4.93
	Medial tibial	**49.44** **±** **9.54**^+^	41.52 ± 8.56	45.70 ±11.85	**51.40** **±** **10.22**^+^	**56.41** **±** 7.63^*^	**50.08** **±** **9.47**^+^
Tb.Th (mm)	Lateral femur	0.429 ± 0.126	0.399 ± 0.177	0.447 ± 0.208	0.300 ± 0.121	0.340 ± 0.140	0.284 ± 0.071
	Medial femur	0.431 ± 0.180	0.448 ± 0.208	0.304 ± 0.057	0.479 ± 0.263	0.470 ± 0.218	**0.465** **±** **0.196**^**+**^
	Lateral tibia	0.303 ± 0.075	0.260 ± 0.900	0.315 ± 0.111	0.371 ± 0.151	0.337 ± 0.063	0.331 ± 0.126
	Medial tibial	0.354 ± 0.068	0.407 ± 0.170	0.277 ± 0.123	0.325 ± 0.069	0.336 ± 0.114	0.364 ± 0.099
Tb.Sp (mm)	Lateral femur	0.425 ± 0.074	0.476 ± 0.192	0.420 ± 0.180	0.426 ± 0.202	0.360 ± 0.128	0.535 ± 0.109
	Medial femur	0.423 ± 0.149	0.438 ± 0.199	0.344 ± 0.085	0.415 ± 0.141	0.439 ± 0.117	0.389 ± 0.155
	Lateral tibia	0.222 ± 0.116	0.301 ± 0.093	0.242 ± 0.113	0.179 ± 0.077	0.223 ± 0.069	0.237 ± 0.073
	Medial tibial	0.403 ± 0.245	0.404 ± 0.182	0.432 ± 0.244	**0.441** **±** **0.162**^**+**^	0.240 ± 0.083	0.352 ± 0.171
Tb.N (1/mm)	Lateral femur	3.000 ± 0.632	3.125 ± 0.641	3.286 ± 0.756	3.429 ± 1.272	4.167 ± 0.983	3.625 ± 0.518
	Medial femur	3.286 ± 0.951	3.750 ± 1.282	3.167 ± 1.329	3.286 ± 0.951	3.375 ± 0.744	3.250 ± 1.035
	Lateral tibia	4.143 ± 0.690	3.875 ± 0.641	4.000 ± 0.926	3.833 ± 0.983	3.875 ± 0.641	3.571 ± 0.535
	Medial tibial	**3.000** **±** **0.816**^+^	**2.375** **±** **0.518**^+^	**2.750** **±** **1.035**^+^	2.833 ± 0.408	**3.857** **±** 0.900^*^	3.250 ± 1.035

Histomorphometric scores of undecalcified samples: Tb.A, trabecular area; Tb.Th, trabecular thickness; Tb:Sp, trabecular separation; Tb:N, trabecular number; Values represent the mean and SD. Statistical significant differences are marked in “bold text.” p < 0.05:

a *vs. SHAM*,

+ *vs. lateral compartment*,

* *vs. OA joints*.

### Micro-Computed Tomography Results

Micro-CT trabecular data of the structural parameters evaluated in the three VOIs selected (central, anterior and posterior) are available on Supplementary Tables 1–3, respectively.

The main statistical differences in the most of the analyzed parameters were found in the OA joints (CONT-OA and RIS-OA) compared to the HT contralateral limbs showing decreased BV/TV, Tb.Th and Tb.N, as well as increased Tb.Sp. Regarding the surgery control groups, although there were hardly any statistical differences between SHAM-HT and SHAM-OA, slightly higher osteoarthritic trabecular changes were identified in the latter. Overall, no significant differences were found between lateral and medial sides in almost any of the parameters analyzed, nor between femoral and tibial compartments.

**Figure 5:**
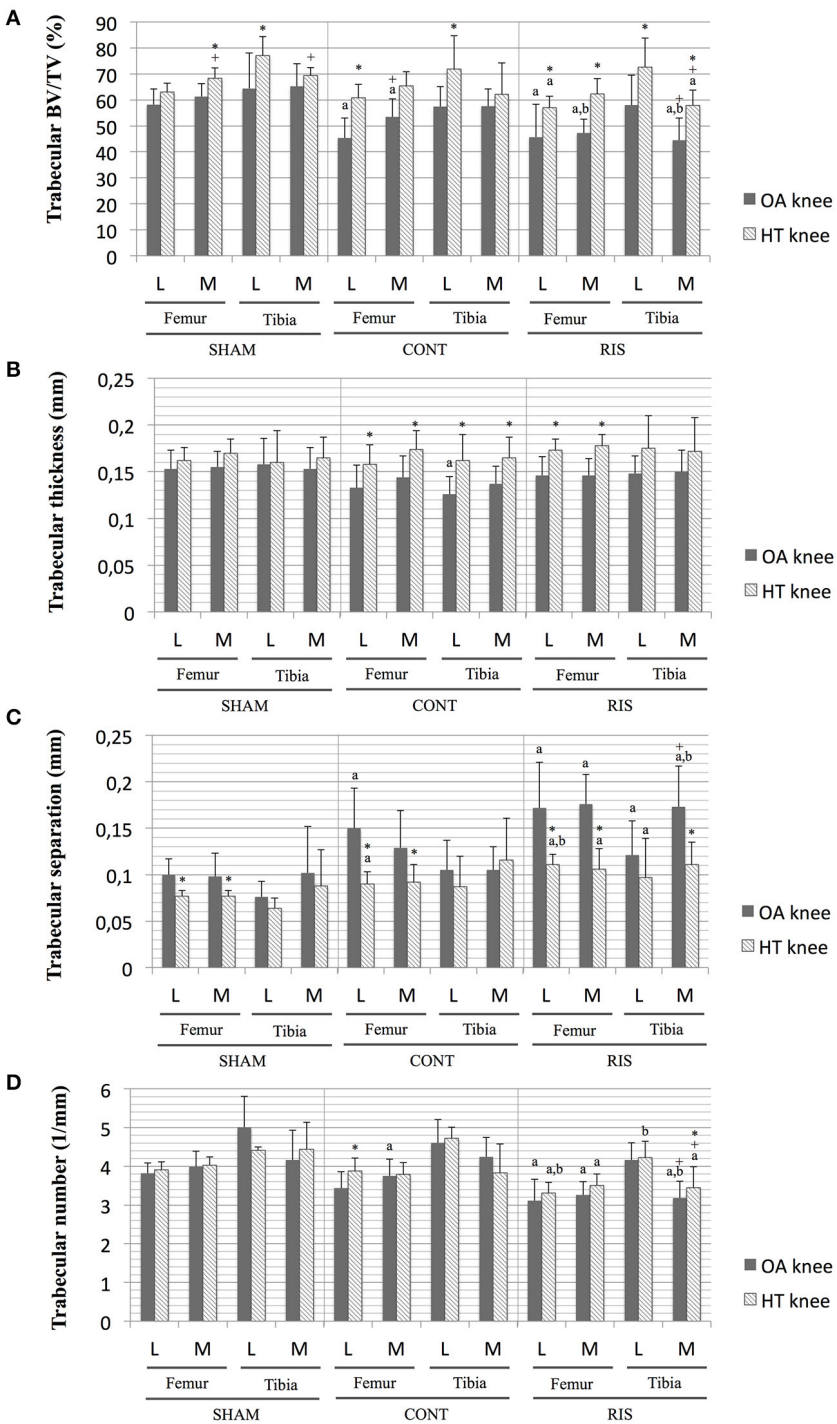
Micro-CT trabecular analysis on central VOI of osteoarthritic and healthy joints. **(A)** BV/TV, bone volumetric fraction; **(B)** Tb.Th, trabecular thickness; **(C)** Tb:Sp, trabecular separation; **(D)** Tb:N, trabecular number. Values given as mean ± SD. Statistical significant differences *p* < 0.05: ^a^vs. SHAM, ^b^vs. CONT, ^+^vs. lateral compartment, *vs. OA joints.

Additionally, in the risedronate-treated animals, altered trabecular bone properties were identified in both HT and OA joints. A significant increase Tb.Sp was observed when compared to the SHAM groups in femoral and tibial compartments, and occasionally, even to the CONT animals. Regarding the Tb.N, the risedronate-treated groups (RIS-OA and RIS-HT) generally showed the lowest values, with significant differences when comparing them to the SHAM OA and HT groups, respectively (Figure 5).

With regard to the relationship between the trabecular parameters obtained by micro-CT and undecalcified histology (Table 5), significant correlations were found for Tb.Sp (*r* = 0.221, *p* < 0.001) and Tb.N (*r* = 0.210, *p* < 0.001). However, these associations were not strongly correlated. Regarding the BV/TV measurements from micro-CT, they were moderately positive correlated with the histomorphometric Tb.A (*r* = 0.437, *p* < 0.001). By contrast, there was no correlation for the Tb.Th assessments (*r* = 0.026, *p* = 0.74).

**Table 5:** Mean values and correlation of Pearson coefficients between 2D histology and 3D micro-CT trabecular parameters.

**Correlation between:**	**2D values (mean ± SD)**	**3D values (mean ± SD)**	***r***	***P*-value**
Tb.A and BV/TV (%)	50.07 ± 9.33	60.09 ± 11.46	0.437	<0.001
Tb.Th (mm)	0.373 ± 0.154	0.156 ± 0.026	0.026	0.74
Tb.Sp (mm)	0.362 ± 0.169	0.109 ± 0.042	0.221	<0.001
Tb.N (1/mm)	3.417 ± 0.938	3.905 ± 0.634	0.210	<0.001

*Tb.A, trabecular area; BV/TV, bone volume fraction; Tb.Th, trabecular thickness; Tb.Sp, trabecular separation; Tb.N, trabecular number*.

## Discussion

The present study studied the histomorphometric quantitative effects of risedronate on cartilage and subchondral bone in an experimental rabbit model of OA after 6 months of treatment. This stereologic analysis may contribute to a better evaluation of the long-term therapeutic effect of bisphosphonates as disease-modifying drugs for OA, also contributing to a better understanding of the typical histological structural alterations in rabbits as a preclinical OA model in an advanced stage.

Surgically induced models have some advantages, compared to spontaneous models, such as a rapid onset and lower costs (48). Nevertheless, the morphological changes are also usually more severe and a minor therapeutic response could be observed, which needs to be taken into account when the experiment is designed (25). Concerning the rabbit as an experimental animal model, although their histological osteoarthritic lesions have been extensively studied by the scientific community for numerous years (29, 33, 42), at present, there are still some differences in the parameters analyzed between preclinical studies. With reference to the anatomical location of the pathological changes, Huang et al. (49) pointed out that at 16 weeks after surgery, rabbits showed partial or full thickness cartilage erosion, more marked in the MFC. These results were consistent with the results of other researches, which reported more pronounced degenerative bone changes in the femur and in the medial compartment (33, 42). They were similar to those observed in advanced human OA where the disease was described as more severe in the medial compartment than in the lateral one (50). In accordance with these findings, a recently published study (45) observed that the medial femoral condyles exhibited slightly greater macroscopic osteoarthritic changes. By contrast, in this study, more structural changes were observed in the cartilage and subchondral bone in terms of thickness values of the MTP. However, one should take into account that significantly higher thickness values were also observed in HT joints, thus an increased thickness at that level could be physiologically present in the ACLT rabbit model.

Regarding other preclinical models such as spontaneous OA in guinea pig, authors also noticed more severe cartilage OA changes on medial sides, as well as on the anterior region (2). Moreover, Bagi et al. (51) observed that in contrast to biped animals, rat models of OA tend to shift part of the weight onto their front legs and not only onto the contralateral limb as humans do. This observation was in agreement with a previous publication, where no restabilization of the damaged limb to the healthy was observed in rodents (25). Nevertheless, in the experimental design it was important to include non-surgically induced control animals (SHAM group), in order to allow the identification of possible load-bearing alterations toward the healthy limb (42). In our study, the histomorphometric assessments showed that the experimental surgical model induced degenerative changes on the articular cartilage and the periarticular subchondral bone and additionally, significant differences were found between the OA joints and the contralateral HT joints in most of the analyzed parameters.

As previously discussed, one of the objectives of this study was to elucidate whether these antiresorptive therapies could somehow influence the OA progression, with special interest in the subchondral bone response. Despite the fact that osteoarthritis investigations should include all tissues that make up the joint, articular cartilage generally continues to be the primary focus of OA (34, 52, 53). To provide a wide understanding of the osteoarthritic disease, various diagnostic techniques may be necessary in preclinical research to properly analyse all the synovial joint elements. Regarding, the new proteomic studies are proving to be an interesting and powerful tool for OA pathophysiology research (54). For the present study, we used quantitative 2D histological histomorphometry to assess the structural changes in cartilage and the subchondral bone properties as well as 3D micro-CT scanning in order to complete the trabecular bone characterization at different anatomical locations.

Within the possible assessable histomorphometric parameters, the cartilage thickness is the most widely evaluated parameter in OA animal models and probably, one of the easiest to quantify (32). Nevertheless, scientific publications showed a great heterogeneity in their outcomes. On the one hand, a cartilage surface degradation during OA progression was described (36, 53, 55), but other studies was observed the increase and the hypertrophy of the cartilage layers (2, 38, 39). In accordance with the latter, we noticed a marked tendency toward cartilage thickening in OA joints in all the anatomical sections examined and these outcomes were especially pronounced in the MTP. By contrast, Pinamont et al. (36) observed a decrease in total cartilage and uncalcified cartilage areas in a surgical mouse model 12 weeks after the OA induction, probably related to cartilage fibrillation and even the complete degeneration of the cartilage layer, during severe OA. In the same way, Hayami et al. (53) found a significant uncalcified cartilage thinning in the MTP in two surgically induced rat models, within 4–10 weeks after surgery. Additionally, Hagiwara et al. (56) did not noticed any significant differences in articular cartilage thickness using an immobilization rat model. This lack of consistent results regarding the cartilage thickness was related to the absence of measurement protocols, the animal model and the use of contralateral knees as controls (56). Concerning the latter, in our study no statistical differences were identified between healthy joints in any of the studied groups (including de SHAM-HT group) suggesting the validity of the contralateral limb used as HT control. As far as the BP response is concerned, in a previous study of our research group (16) it was observed that short-term risedronate therapy seemed to prevent the cartilage thickening, showing similar values to those observed in the healthy control animals. However, in this instance, we did not observe this capacity in the long term and no statistical differences were found compared to the placebo group.

Regarding the pathological changes in bone tissues, a noticeable relationship between damaged cartilage and underlying subchondral bone alteration was observed according to several publications (2, 39, 51, 57). In concordance with this, our analysis showed a trend toward subchondral bone thinning beneath the cartilage alteration. By contrast, other publications noticed a thicker subchondral bone layer in the osteoarthritic joints (38, 57). In early or less severe stages of OA, Wang et al. (2) suggested that both cartilage and subchondral bone tissue may initially respond by an increasing remodeling activity which would be manifested as an uncalcified cartilage layer increase. Conversely, the initial response of the subchondral bone to mechanical stress could increase the osteoclasts activity resulting in a thinner subchondral bone plate. Although we did not find any significant differences in the SB.Th of femoral condyles between the risedronate- and placebo-treated groups, we observed that tibial plateaus with a long-term risedronate administration showed slightly higher thickness values, in both OA and HT joints, compared to untreated animals. This occurrence could be associated to an inhibition of bone-resorption activity at this level, related to the BP administration.

The present study also evaluated the FI, which is considered a histopathological indicator of collagen breakdown in OA (55). Additionally, Hayami et al. described that focal fibrillation was one of the first degenerative changes observed in the rat surgical model (53). BP therapies demonstrated significant chondroprotective effects in preclinical studies, showing significantly milder ulcerations and less superficial cartilage fibrillation (16, 58). By contrast, in our assessments after 6 months of treatment, risedronate did not seem to improve the cartilage pathological changes, showing similar values to those observed in untreated animals. In accordance with other publications (35), the OA joints showed significant more surface fibrillation than HT joints, especially in the medial compartments. However, a recent research study has highlighted that although this parameter may be indicative of the severity of the cartilage, it may also decrease due to a complete erosion of the cartilage surface in advanced stages of OA (36).

Regarding the results obtained in the trabecular micro-CT analysis, the main changes were detected in the osteoarthritic joints compared to the healthy ones, and were as follows: reduced BV/TV, smaller Tb.Th and Tb.N, as well as increased Tb.Sp. Our findings were consistent with other post-traumatic studies using the ACLT rabbit model (33, 42) and canine model (41), where it was observed an increased deterioration in the quality of the trabecular bone with activated bone resorption and consequently, decreased Tb.Th and bone volume. In contrast to other publications, where increased trabecular bone loss was observed on femoral condyles against tibial plateaus (33), overall in our study, no significant differences between femoral and tibial compartments were found in any of the VOIs analyzed. In that regard, contradictory results were found between scientific studies based on animal models and the experimental protocol. Interestingly, and contrary to what it has been published in some preclinical models, Bobinac et al. (50) observed in human knee joints with osteoarthritis, a significant histomorphometry increase in BV/TV and Tb.Th parameters, as well as decreased values of Tb.N and Tb.S. As proposed by Chappard et al. (59) the differences between human OA studies and those observed in animal models, may be related to variations between spontaneous and post-traumatic OA. Additionally, other factors such as the presence of comorbid diseases should be take into account and have been related with significant bone loss at the subchondral plate. Specifically, it has been observed in human knee OA patients with type 2 diabetes mellitus and hypertension (60). With respect to the risedronate therapy effectiveness, although several preclinical publications seemed to show a positive subchondral bone preservation (16, 17, 23), in our case, the long-term BP administration did not show any beneficial effect on the osteoarthritic joints, showing lower Tb.N and higher Tb.Sp values, similar to those observed in the CONT-OA group. Interestingly, similar altered trabecular bone properties were noticed in RIS-HT joints, and more specifically it was observed in the central VOI. This could be explained due to the inherent antiresorptive properties of these therapies, but it could also be related to an altered load-bearing on the contralateral healthy limb, showing statistical differences when compared to the SHAM-HT group.

As other authors noticed (61), bone histomorphometry presented some limitations associated with its 2D condition and the slice selection and size, representing only a very small fraction of the synovial tissue. The correlations observed between 2D histomorphometry and micro-CT measurements were weak, and there were hardly any significant differences in the histological trabecular analysis among groups. By contrast, micro-CT evaluation identified more accurate changes in the cancellous bone microarchitecture.

The alterations observed in both cartilage and subchondral bone reflected the concomitant response of those articular tissues to the surgical instability and consequently, to the altered biomechanical loads. As expected from increased focal load-bearing associated with surgical location (medial meniscectomy), cartilage tissues in medial compartments showed an increased response to overloading, especially on the tibial compartment. This is consistent with previous publications, where a faster progression of OA was observed in the tibial plateau compared to femoral condyles (39). Nevertheless, in that sense we could expect a more increased pathology in the middle zones (Z2 and Z3) of femorotibial joints, but the measurements in femoral condyles were too variable to establish the hypothesis. With respect to tibial plateaus, the highest values were identified in Z1 and Z2, corresponding to the inner zones.

Concerning the general BP effectiveness, in our study the long-term risedronate administration did not seem to reduce the osteoarthritic changes and failed to diminish the hypertrophic response of cartilage, the surface fibrillation or prevent the subchondral trabecular bone loss. Previous publications noted that pre-emptive and early antiresorptive therapy initiations showed better chondroprotective efficacy and subchondral bone quality than delayed administrations (14, 62, 63). It is therefore possible that our rabbit ACLT and partial medial meniscectomy model developed the cartilage damage too fast and when we started the risedronate administration, the observed changes were already too severe. However, the real situation in affected patients, in both human and veterinary medicine, is that therapies are usually initiated when the OA disease is advanced (14). For this reason, early diagnostic techniques, such as new imaging modalities and biochemical markers should provide a new opportunity for the future management of the disease.

Our study is subject to several limitations. First, our analysis included only 24 animals and there may be variability differences of some of the parameter measures. Additionally, some technical challenges should be kept in mind, such as the high cost, the highly trained personnel required and the slide selection, which may not occur consistently. Lastly, another potential limitation of the current study is that the histological techniques may not be completely applicable in clinical situations.

In conclusion, it is important to note that the scientist community is currently involved in carrying out the 3R principles of replacement, reduction and refinement. Given that we adhered to a policy of reducing animal numbers, we considered to be appropriate to continue the research with the same experimental animals (45) and consequently, complete the characterization of the risedronate effect at all levels. The results of this study may improve our knowledge about osteoarthritis anatomic location and disease evolution, more specifically allowing an appropriate tissue selection in the OA preclinical studies. Additionally, this study provides novel information about the histomorphometry quantitative effect of long-term bisphosphonate use as possible disease-modifying drug in osteoarthritis.

## Conclusions

In this study, the histomorphometric evaluations in the rabbit surgical model were appropriate for the study of the quantitative structural osteoarthritic changes, contributing to a better evaluation of the long-term therapeutic efficacy of bisphosphonates and also, to a better anatomical knowledge of the animal model. Sample analysis demonstrated that the experimental model induced osteoarthritic changes in the operated joints. The histomorphometric analysis of the undecalcified histology samples showed a marked tendency toward cartilage thickening and cartilage fibrillation in the OA joints in all the examined anatomical sections, as well as a trend toward subchondral bone thinning beneath the cartilage alteration. These outcomes were especially pronounced in the medial tibial plateau and in the inner tibial zones. Additionally, the trabecular evaluation showed a significant bone quality loss in the operated limbs with decreased trabecular thickness, trabecular number and bone volume, as well as increased trabecular separation. The micro-CT analysis identified more accurately the trabecular bone changes than the undecalcified histology. Lastly, the long-term risedronate treatment did not seem to have the capacity to reduce the osteoarthritic hypertrophic cartilage response and failed to diminish the surface fibrillation or prevent the trabecular bone loss.

## Data Availability Statement

The original contributions presented in the study are included in the article/Supplementary Material, further inquiries can be directed to the corresponding author/s.

## Ethics Statement

The animal study was reviewed and approved by Ethical Committee of University of Santiago de Compostela (Spain) (protocol code 01/16/LU-002, approved on March 18, 2016).

## Author Contributions

AG-C, ML-P, and FM conceived, designed, and supervised the study. SF-M, AG-C, ML-P, MP, and FM undertook the experimental work. Histological analyses were made by SF-M and MG-G. Micro-CT evaluation was performed by MP. SF-M analyzed the data and drafted the manuscript. AG-C, ML-P, MP, MG-G, and FM revised and editing the final manuscript. All authors have read and approved the submitted version of the manuscript.

## Conflict of Interest

The authors declare that the research was conducted in the absence of any commercial or financial relationships that could be construed as a potential conflict of interest.
