# Supplementary material for: Histomorphometric Quantitative Evaluation of Long-Term Risedronate Use in a Knee Osteoarthritis Rabbit Model
Source: Front Vet Sci. 2021 Apr 22;8:669815. doi: 10.3389/fvets.2021.669815 (PMC8100024; doi:10.3389/fvets.2021.669815)
Supplement: Supplementary file 1 [file Data_Sheet_1.PDF]

Supplementary Table 1. Subchondral trabecular bone Micro-CT analysis on central VOI of OA and healthy joints.

		**Osteoarthritis joints (OA)**			**Healthy joints (HT)**		
		**SHAM**	**CONT**	**RIS**	**SHAM**	**CONT**	**RIS**
**BV/TV (%)**	**Lateral femur**	58.21 ± 5.97	**45.36 ± 7.74** [a]	**45.71 ± 12.63** [a]	63.11 ± 3.28	**60.79 ± 5.18** [*]	**57.01 ± 4.48** [a, *]
	**Medial femur**	61.33 ± 4.97	**53.43 ± 6.99** [a, +]	**47.29 ± 5.34** [a, b]	**68.32 ± 4.07** [*, +]	65.45 ± 5.43	**62.27 ± 5.98** [*]
	**Lateral tibia**	64.46 ± 13.64	57.45 ± 7.74	58.06 ± 11.47	**77.14 ± 7.28** [*]	**71.88 ± 12.89** [*]	**72.71 ± 11.2** [*]
	**Medial tibia**	65.29 ± 8.75	57.61 ± 6.59	**44.48 ± 8.6** [a, b, +]	**69.47 ± 3.05** [+]	62.22 ± 12.09	**57.96 ± 5.79** [a, *, +]
**Tb.Th (mm)**	**Lateral femur**	0.153 ± 0.020	0.133 ± 0.024	0.146 ± 0.020	0.162 ± 0.014	**0.158 ± 0.021** [*]	**0.173 ± 0.012** [*]
	**Medial femur**	0.155 ± 0.017	0.144 ± 0.023	0.146 ± 0.018	0.170 ± 0.015	**0.174 ± 0.020** [*]	**0.178 ± 0.012** [*]
	**Lateral tibia**	0.158 ± 0.028	**0.126 ± 0.019** [a]	0.148 ± 0.019	0.160 ± 0.034	**0.162 ± 0.028** [*]	0.175 ± 0.035
	**Medial tibia**	0.153 ± 0.023	0.137 ± 0.019	0.150 ± 0.023	0.165 ± 0.022	**0.165 ± 0.022** [*]	0.172 ± 0.036
**Tb.Sp (mm)**	**Lateral femur**	0.100 ± 0.017	**0.150 ± 0.043** [a]	**0.172 ± 0.049** [a]	**0.077 ± 0.006** [*]	**0.090 ± 0.013** [a, *]	**0.111 ± 0.011** [a, b, *]
	**Medial femur**	0.098 ± 0.025	0.129 ± 0.040	**0.176 ± 0.032** [a]	**0.077 ± 0.006** [*]	**0.092 ± 0.019** [*]	**0.106 ± 0.022** [a, *]
	**Lateral tibia**	0.076 ± 0.017	0.105 ± 0.032	**0.121 ± 0.037** [a]	0.064 ± 0.011	0.087 ± 0.033	**0.097 ± 0.042** [a]
	**Medial tibia**	0.102 ± 0.050	0.105 ± 0.025	**0.173 ± 0.044** [a, b, +]	0.088 ± 0.039	0.116 ± 0.045	**0.111 ± 0.024** [*]
**Tb.N (1/mm)**	**Lateral femur**	3.822 ± 0.269	3.436 ± 0.421	**3.110 ± 0.558** [a]	3.914 ± 0.204	**3.884 ± 0.330** [*]	**3.307 ± 0.276** [a, b]
	**Medial femur**	3.993 ± 0.398	**3.752 ± 0.434** [a]	**3.260 ± 0.344** [a]	4.031 ± 0.212	3.795 ± 0.300	**3.502 ± 0.301** [a]
	**Lateral tibia**	5.012 ± 0.796	4.605 ± 0.604	4.166 ± 0.441	4.415 ± 0.084	4.725 ± 0.29	**4.223 ± 0.427** [b]
	**Medial tibia**	4.165 ± 0.766	4.241 ± 0.499	**3.181 ± 0.430** [a,b,+]	4.442 ± 0.695	3.835 ± 0.744 [+]	**3.449 ± 0.537** [a, *, +]

Micro-CT results: BV/TV: bone volumetric fraction; Tb.Th: trabecular thickness; Tb:Sp: trabecular separation; Tb:N: trabecular number. Values represent the mean and SD. Statistical significant differences are marked in “bold text.” $p< 0.05$: [a] vs. SHAM, [b] vs. CONT, [+] vs. lateral compartment, [*] vs. OA joints
