# Supplementary material for: Histomorphometric Quantitative Evaluation of Long-Term Risedronate Use in a Knee Osteoarthritis Rabbit Model
Source: Front Vet Sci. 2021 Apr 22;8:669815. doi: 10.3389/fvets.2021.669815 (PMC8100024; doi:10.3389/fvets.2021.669815)
Supplement: Supplementary file 2 [file Data_Sheet_2.PDF]

Supplementary Table 2. Subchondral trabecular bone Micro-CT analysis of anterior VOI on OA and healthy joints.

		Osteoarthritis joints (OA)			Healthy joints (HT)		
		**SHAM**	**CONT**	**RIS**	**SHAM**	**CONT**	**RIS**
**BV/TV (%)**	**Lateral femur**	65.99 ± 5.35	54.52 ± 7.78	56.76 ± 11.98	**71.96 ± 3.66** [*]	**70.13 ± 4.50** [*]	**66.88 ± 4.02** [*]
	**Medial femur**	73.85 ± 4.96	59.42 ± 9.51	**57.07 ± 4.96** [a,b]	74.73 ± 4.53	**73.82 ± 5.17** [*]	**71.57 ± 4.57** [*]
	**Lateral tibia**	67.83 ± 13.99	53.27 ± 9.98	55.75 ± 11.67	76.19 ± 10.55	**73.55 ± 10.18** [*]	**73.30 ± 9.15** [*]
	**Medial tibia**	67.65 ± 13.66	**62.99 ± 5.48** [+]	55.67 ± 11.71	75.12 ± 12.00	72.87 ± 11.48	**70.66 ± 9.40** [*]
**Tb.Th (mm)**	**Lateral femur**	0.157 ± 0.021	0.140 ± 0.022	0.159 ± 0.018	0.173 ± 0.022	**0.170 ± 0.019** [*]	**0.186 ± 0.016** [*]
	**Medial femur**	0.166 ± 0.016	0.144 ± 0.023	0.148 ± 0.015	0.174 ± 0.017	**0.178 ± 0.021** [*]	**0.189 ± 0.017** [*]
	**Lateral tibia**	0.146 ± 0.024	**0.115 ± 0.015** [a]	0.136 ± 0.022	0.165 ± 0.019	**0.162 ± 0.031** [*]	**0.170 ± 0.029** [*]
	**Medial tibia**	0.163 ± 0.020	**0.138 ± 0.023** [+]	0.140 ± 0.027	0.178 ± 0.023	0.176 ± 0.042	**0.192 ± 0.0431** [*, +]
**Tb.Sp (mm)**	**Lateral femur**	0.092 ± 0.016	0.117 ± 0.019	0.120 ± 0.026	**0.073 ± 0.006** [*]	**0.083 ± 0.012** [*]	**0.100 ± 0.013** [a, b]
	**Medial femur**	0.076 ± 0.0134	**0.108 ± 0.017** [a]	**0.134 ± 0.023** [a,b]	0.079 ± 0.015	**0.082 ± 0.013** [*]	**0.098 ± 0.012** [a, b, *]
	**Lateral tibia**	0.076 ± 0.014	0.096 ± 0.022	**0.120 ± 0.028** [a]	0.063 ± 0.012	0.080 ± 0.026	**0.089 ± 0.026** [*]
	**Medial tibia**	0.078 ± 0.014	0.091 ± 0.017	**0.139 ± 0.041** [a]	0.069 ± 0.018	0.088 ± 0.028	**0.089 ± 0.022** [*]
**Tb.N (1/mm)**	**Lateral femur**	4.224 ± 0.294	3.917 ± 0.424	**3.553 ± 0.499** [a]	4.190 ± 0.349	4.160 ± 0.357	**3.603 ± 0.250** [a, b]
	**Medial femur**	4.483 ± 0.395	4.144 ± 0.423	**3.681 ± 0.357** [a]	4.317 ± 0.325	4.187 ± 0.472	**3.793 ± 0.243** [a]
	**Lateral tibia**	4.983 ± 0.380	4.676 ± 0.803	**4.053 ± 0.596** [a]	4.872 ± 0.590	4.795 ± 0.471	4.382 ± 0.577
	**Medial tibia**	4.486 ± 0.606	4.628 ± 0.523	3.996 ± 0.680	4.526 ± 0.624	4.606 ± 0.858	3.814 ± 0.754

Micro-CT results: BV/TV: bone volumetric fraction; Tb.Th: trabecular thickness; Tb:Sp: trabecular separation; Tb:N: trabecular number. Values represent the mean and SD. Statistical significant differences are marked in "bold text." $p< 0.05$: [a] vs. SHAM, [b] vs. CONT, [+] vs. lateral compartment, [*] vs. OA joints
