# Supplementary material for: Histomorphometric Quantitative Evaluation of Long-Term Risedronate Use in a Knee Osteoarthritis Rabbit Model
Source: Front Vet Sci. 2021 Apr 22;8:669815. doi: 10.3389/fvets.2021.669815 (PMC8100024; doi:10.3389/fvets.2021.669815)
Supplement: Supplementary file 3 [file Data_Sheet_3.PDF]

Supplementary Table 3. Subchondral trabecular bone Micro-CT analysis on posterior VOI of OA and healthy joints.

		**Osteoarthritis joints (OA)**			**Healthy joints (HT)**		
		**SHAM**	**CONT**	**RIS**	**SHAM**	**CONT**	**RIS**
**BV/TV (%)**	**Lateral femur**	70.42 ± 2.98	**54. 73 ± 9.09** [a]	**53.47 ± 10.29** [a]	73.74 ± 3.55	**72.94 ± 4.18** [*]	**74.14 ± 3.73** [*]
	**Medial femur**	68.88 ± 2.52	61.02 ± 8.33	**53.90 ± 7.70** [a]	72.30 ± 6.18	**72.29 ± 4.61** [*]	**70.06 ± 5.66** [*, +]
	**Lateral tibia**	70.88 ± 8.48	58.82 ± 13.08	**53.84 ± 7.47** [a]	73.67 ± 13.70	70.74 ± 11.70	**71.08 ± 6.88** [*]
	**Medial tibia**	70.55 ± 4.26	63.68 ± 9.03	62.28 ± 11.38	72.18 ± 12.66	68.78 ± 10.22	66.72 ± 9.26
**Tb.Th (mm)**	**Lateral femur**	0.174 ± 0.019	**0.137 ± 0.024** [a]	0.158 ± 0.026	0.185 ± 0.010	**0.183 ± 0.029** [*]	**0.204 ± 0.016** [*]
	**Medial femur**	0.170 ± 0.015	0.149 ± 0.018	0.157 ± 0.026	0.178 ± 0.018	**0.194 ± 0.025** [*]	**0.198 ± 0.025** [*]
	**Lateral tibia**	0.144 ± 0.025	0.125 ± 0.026	0.132 ± 0.017	0.159 ± 0.015	0.151 ± 0.030	0.151 ± 0.037
	**Medial tibia**	0.152 ± 0.016	0.145 ± 0.016	**0.165 ± 0.032** [+]	0.162 ± 0.005	0.162 ± 0.035	0.170 ± 0.033
**Tb.Sp (mm)**	**Lateral femur**	0.082 ± 0.007	**0.127 ± 0.039** [a]	**0.147 ± 0.034** [a]	**0.071 ± 0.005** [*]	**0.078 ± 0.007** [*]	**0.086 ± 0.005** [a, b, *]
	**Medial femur**	0.081 ± 0.008	0.116 ± 0.041	**0.141 ± 0.027** [a]	0.077 ± 0.013	**0.082 ± 0.013** [*]	**0.094 ± 0.012** [*]
	**Lateral tibia**	0.085 ± 0.033	0.101 ± 0.033	**0.142 ± 0.050** [a]	0.067 ± 0.016	0.094 ± 0.033	**0.080 ± 0.011** [*]
	**Medial tibia**	0.085 ± 0.026	0.089 ± 0.019	**0.138 ± 0.049** [a]	0.073 ± 0.021	0.101 ± 0.038	**0.089 ± 0.014** [*]
**Tb.N (1/mm)**	**Lateral femur**	4.063 ± 0.297	3.746 ± 0.767	3.414 ± 0.532	3.995 ± 0.219	4.055 ± 0.471	**3.544 ± 0.270** [a, b]
	**Medial femur**	4.072 ± 0.360	3.951 ± 0.603	3.459 ± 0.363	4.080 ± 0.176	3.779 ± 0.443	**3.569 ± 0.308** [a]
	**Lateral tibia**	4.968 ± 0.321	4.398 ± 0.440	**4.102 ± 0.518** [a]	4.718 ± 0.137	4.788 ± 0.269	4.365 ± 0.451
	**Medial tibia**	4.673 ± 0.459	4.453 ± 0.871	3.802 ± 0.527	4.751 ± 0.311	4.555 ± 0.699	4.197 ± 0.765

Micro-CT results: BV/TV: bone volumetric fraction; Tb.Th: trabecular thickness; Tb:Sp: trabecular separation; Tb:N: trabecular number. Values represent the mean and SD. Statistical significant differences are marked in "bold text." $p < 0.05$: [a] vs. SHAM, [b] vs. CONT, [+] vs. lateral compartment, [*] vs. OA joints
